# Dengue and yellow fever virus vectors: seasonal abundance, diversity and resting preferences in three Kenyan cities

**DOI:** 10.1186/s13071-017-2598-2

**Published:** 2017-12-29

**Authors:** Sheila B. Agha, David P. Tchouassi, Armanda D. S. Bastos, Rosemary Sang

**Affiliations:** 1 International Centre of Insect Physiology and Ecology, P. O Box 30772-00100, Nairobi, Kenya; 2 Department of Zoology and Entomology, University of Pretoria, Private Bag 20, Hatfield, 0083 South Africa; 3 Arbovirus/Viral Hemorrhagic Fever Laboratory, Centre for Virus Research, Kenya Medical Research Institute, P. O Box 54840-00200, Nairobi, Kenya

**Keywords:** *Aedes aegypti*, *Aedes bromeliae*, Vector abundance, Mosquito diversity, Resting preference, Urbanization, Kenya, Dengue and yellow fever risk

## Abstract

**Background:**

The transmission patterns of dengue (DENV) and yellow fever (YFV) viruses, especially in urban settings, are influenced by *Aedes* (*Stegomyia*) mosquito abundance and behavior. Despite recurrent dengue outbreaks on the Kenyan coast, these parameters remain poorly defined in this and other areas of contrasting dengue endemicity in Kenya. In assessing the transmission risk of DENV/YFV in three Kenyan cities, we determined adult abundance and resting habits of potential *Aedes* (*Stegomyia*) vectors in Kilifi (dengue-outbreak prone), and Nairobi and Kisumu (no dengue outbreaks reported). In addition, mosquito diversity, an important consideration for changing mosquito-borne disease dynamics, was compared.

**Methods:**

Between October 2014 and June 2016, host-seeking adult mosquitoes were sampled using CO_2_-baited BG-Sentinel traps (12 traps daily) placed in vegetation around homesteads, across study sites in the three major cities. Also, indoor and outdoor resting mosquitoes were sampled using Prokopack aspirators. Three samplings, each of five consecutive days, were conducted during the long-rains, short-rains and dry season for each city. Inter-city and seasonal variation in mosquito abundance and diversity was evaluated using general linear models while mosquito-resting preference (indoors *vs* outdoors) was compared using Chi-square test.

**Results:**

*Aedes aegypti*, which comprised 60% (*n* = 7772) of the total 12,937 host-seeking mosquitoes collected, had comparable numbers in Kisumu (45.2%, *n* = 3513) and Kilifi (37.7%, *n* = 2932), both being significantly higher than Nairobi (17.1%, *n* = 1327). *Aedes aegypti* abundance was significantly lower in the short-rains and dry season relative to the long-rains (*P* < 0.0001). *Aedes bromeliae*, which occurred in low numbers, did not differ significantly between seasons or cities. Mosquito diversity was highest during the long-rains and in Nairobi. Only 10% (*n* = 43) of the 450 houses aspirated were found positive for resting *Ae. aegypti*, with overall low captures in all areas. *Aedes aegypti* densities were comparable indoors/outdoors in Kilifi; but with higher densities outdoors than indoors in Kisumu and Nairobi.

**Conclusions:**

The presence and abundance of *Ae. aegypti* near human habitations and dwellings, especially in Kilifi/Kisumu, is suggestive of increased DENV transmission risk due to higher prospects of human vector contact. Despite low abundance of *Ae. bromeliae* suggestive of low YFV transmission risk, its proximity to human habitation as well as the observed diversity of potential YFV vectors should be of public health concern and monitored closely for targeted control. The largely outdoor resting behavior for *Ae. aegypti* provides insights for targeted adult vector control especially during emergency outbreak situations.

## Background

Global epidemics of dengue and yellow fever are on the rise in most tropical and subtropical regions, with geographical expansion and increasing frequency of outbreaks being reported, especially in Africa [1–4]. Dengue virus (DENV) is the most rapidly spreading arbovirus in the world, with over 390 million global infections reported yearly [5, 6], whereas yellow fever virus (YFV) has a mortality rate of 20–50%, rivaling that of Ebola virus. Both are arboviral diseases of major public health concern [4].

Since the first dengue outbreak in Kenya in 1982, which occurred in Kilifi and Malindi, subsequent outbreaks have mostly been limited to the Kenyan coast, especially in the urban city of Mombasa [7–9] and recently also affecting the Kenya-Somali border area [10]. This expansion in the geographical range of dengue outbreaks is of concern, as it highlights the potential for further spread. Urban yellow fever outbreaks are on the rise, as recently reported in Angola (Luanda) and Democratic Republic of Congo (Kinshasa), with cases imported into China and Kenya [4, 11, 12]. Although the last yellow fever outbreak in Kenya occurred in 1992–1993 [13], the disease is still considered a public health threat. This is driven in part by the potential for spread through national/international travel [4, 14] as well as the widespread presence of domestic/peri-domestic vectors, *Aedes aegypti* and *Aedes bromeliae* [15].

Yellow fever has continued to re-emerge in the last decade despite the availability of a safe and efficacious vaccine. Although a new dengue vaccine for use in emergency situations in highly endemic countries is currently available [16], the vaccine has not been licensed for use in many endemic countries, including Kenya. Current efforts for controlling dengue in Kenya therefore rely on reducing human-vector contacts as well as continuous suppression of the vector *Ae. aegypti* by targeting the immature stages. Based on previous studies carried out in Kenya, the most productive containers types for *Aedes* immatures were determined for targeted vector control, and the associated *Stegomyia* risk indices were established for assessing risk of DENV/YFV transmission [9, 17]. However, studies focusing on adult mosquito populations are known to be more informative in estimating risk of transmission of these diseases [18]. Also, emergency interventions targeting adults remain crucial during outbreaks, the effectiveness of which depends on a good understanding of the adult abundance and resting behavior.

*Aedes aegypti aegypti* and *Ae. aegypti formosus* (hereafter referred to as *Ae. aegypti*) are genetically diverse forms of *Ae. aegypti*, with the former being highly domesticated and often found in close association with humans, especially in urban settings, as opposed to the more zoophilic genetic form *Ae. aegypti formosus* [19]*.* As reported in large areas of Asia and South America, vectorial capacity of *Ae. aegypti* is influenced to a large extent not only by its extremely high human feeding tendency, but importantly, abundance and indoor resting habits [1, 20], which serve to enhance human-vector contact and maximize disease transmission. Surprisingly, knowledge of these attributes remains poorly defined in major dengue foci of Africa, especially in Kenya.

As part of an epidemiological assessment of risk of dengue and yellow fever outbreaks in Kenya, this study focused on estimating the abundance and diversity of potential DENV/YFV vectors in dengue-endemic (Kilifi County) and dengue-free (Kisumu and Nairobi County) cities in Kenya. We also assessed the domestic and peri-domestic resting habits of potential DENV/YFV vectors in these cities, since the bionomics of a vector is known to shape the epidemiology of the disease*.* Data on the adult abundance and diversity can guide on the level of risk of transmission within each city, while identification of resting habits can be useful in vector control programs, providing baseline information on the different adult mosquito control strategies that can be implemented in case of an epidemic.

## Methods

### Study site

**Fig. 1:**
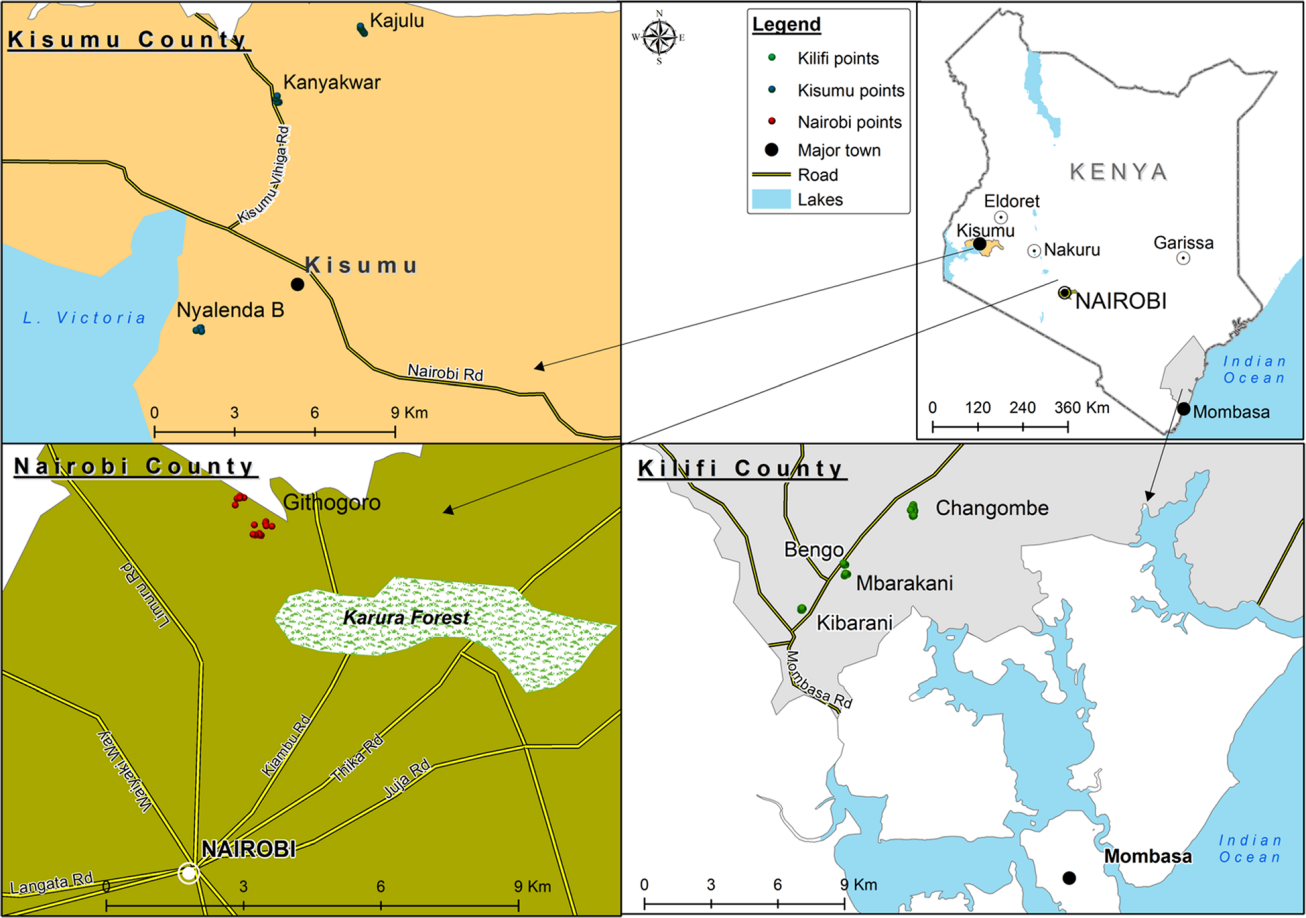
Map showing the location of the study sites within Kilifi, Kisumu and Nairobi Counties of Kenya

The study was carried out in three of the largest cities in Kenya, which despite all being major trade and travel hubs, differ with respect to dengue status with Kilifi being endemic to dengue, whereas there are no reports of dengue from Kisumu and Nairobi (Fig. 1). The capital Nairobi (01°17′S, 36°48′E) is the largest city of Kenya and is located at an altitude of 1661 m above sea level (masl). Average monthly temperature ranges between 22 and 28 °C. Kilifi County, situated at an altitude of 50 masl, occurs within the Coastal Region, incorporating Mombasa (4°03′S, 39°40′E), the second largest city in Kenya. With an average monthly temperature range between 27 and 31 °C, the Coastal Region has been endemic for dengue since 1982, and has experienced recent, as well as recurrent outbreaks in the last decade. Kisumu (00°03′S, 34°45′E), the third largest city in Kenya is second only to Kampala in importance, within the Lake Victoria Region. It is situated at an altitude of 1131 masl and has an average monthly temperature range between 28 and 30 °C.

Traps were set in four sub-locations within Kilifi County in Rabai including Bengo, Changombe, Kibarani, and Mbarakani. Similarly, trapping in Kisumu (Kisumu County), covered three sub-locations- Kajulu, Kanyakwar, and Nyalenda B. In Nairobi (Nairobi County), all traps were set in Githogoro. Sampling at sub-locations, conducted to ensure the widest possible coverage within each city, was balanced against logistical considerations, such as ease of accessibility to homesteads, particularly within Nairobi.

### Study design

Sampling was conducted in the long-rains (April-June), short-rains (October-December) and dry season (January-March or July-September), during 2014–2016. The seasons were primarily defined by the amount of rainfall. We obtained average rainfall data two weeks prior to mosquito sampling from the Kenya Meteorological Department, which during the long-rains was 12.4, 10.8 and 8.3 mm; short-rains 5.5, 4.0 and 7.3 mm and the dry season 0, 0.3 and 0 mm in Kilifi, Kisumu and Nairobi, respectively.

Adult host-seeking mosquitoes were collected using BG-Sentinel traps (BioQuip Products, Rancho Dominguez, CA, USA), baited with CO_2_ supplied in the form of dry ice and placed outdoors in the vegetation around human habitations. The CO_2_ was dispensed by placing ~0.5 kg in a thermos Igloo (2 l) per trap and suspended slightly above the trap entry. Traps (12 daily) were set up at 7 am in the morning and retrieved at 6 pm in the evening on the same day, for 5 consecutive days, in each season in each city, translating to a total of 540 BG-Sentinel traps being set (180 per city and 60 per season).

Collection of resting adult mosquitoes was performed using Prokopack aspirators (BioQuip Products, Rancho Dominguez, CA, USA) targeting *Aedes* resting mosquitoes indoors (sitting room, bedroom and kitchen) and outdoors (on nearby vegetation and the walls outside the house). Houses in each city were purposively selected to include houses with a common design and most importantly availability of surrounding vegetation. Each sampling season targeted a total of 50 houses per city; a total of 450 houses (50 per season and 150 per city) were sampled in all three cities. Sampling was done in the long-rains, short-rains and dry season for 5 consecutive days. Collection was done between 11:00 am to 3:00 pm daily by a team of three people (one indoor and 2 outdoor) lasting 20 min per house.

Trapped mosquitoes were taken to a temporary field site laboratory in each city and immobilized using triethylamine (TAE), placed in cryovials and immediately preserved in liquid nitrogen for transportation to the laboratory at the International Centre of Insect Physiology and Ecology, Nairobi for identification. Morphological identification was done using available taxonomic keys [21–23]. Data on the collection date, species, season and city of collection was captured in Excel. Some mosquitoes that could not be identified to species level, because some of the morphological features were damaged or lost, were classified to genus level as *Aedes* spp., *Mansonia* spp. and *Culex* spp.

### Data analysis

Mosquitoes collected during each of the 5-day trappings per season (i.e. BG-Sentinel collections) from different sites within each city were pooled and counted. We analyzed the total mosquito abundance and specific-species abundance (*Ae. aegypti* and *Ae. bromeliae*) using general linear models (GLMs) with seasons and cities as predictors. As a measure of mosquito community structure, we estimated the Shannon diversity index (hereafter referred to as diversity) for each city per trapping season using the *vegan *package in R version 3.2.3 [24]. We explored seasonal and city influence on mosquito diversity by applying GLMs after log-transformation to normalize the data. Best-fit models (normal or poisson or quasipoisson or negative binomial generalized linear models) were selected based on model residuals for species richness, diversity, total abundance and species-specific abundance. Data normality was confirmed by performing Shapiro-Wilk tests on model residuals of mosquito diversity. Kilifi was taken as the reference city, and the dry season as the reference season.

For resting mosquito collections, we limited our comparisons to *Ae. aegypti* only. Resting *Ae. aegypti* collected for the different seasons in each city were pooled, and broadly classified as indoors (sitting room, bedroom and kitchen) and outdoors (walls around the house and vegetation). The mosquito resting density estimated as the total number of resting *Ae. aegypti* by number of collectors was compared, indoors versus outdoors, for each city using the Chi-square test. The proportion of houses positive indoors or outdoors was also compared using the Chi-square test. All data were analyzed in R version 3.2.3 [24] at α = 0.05 level of significance.

## Results

### Mosquito abundance and composition

A total of 12,937 mosquitoes representing 6 genera and 25 species were captured throughout the survey from the three cities using the BG-Sentinel traps (Table 1). *Aedes aegypti* was the most dominant DENV/YFV vector represented across all the cities and seasons except for Nairobi where *Aedes tricholabis* dominated collections during the long-rains. Kilifi had a wider *Aedes* species representation (9 spp.) and *Mansonia* was primarily encountered in Kisumu, especially in the long-rains and dry season. Collections of *Culex* species were generally low; dominated by *Culex pipiens* and *Culex univittatus* in Kisumu during the long-rains, and wider species representation (10 species) in Nairobi. *Culex rubinotus* was limited to Kilifi, *Culex poicilipes* to Kisumu and *Culex zombaensis* to Nairobi, although in low numbers. *Toxorhynchites brevipalpis* was also recorded in Kilifi and Kisumu, *Eretmapodites chrysogaster* in Kilifi and Nairobi, while *Anopheles* species were recorded in all three areas during the long-rains, although in low numbers.

**Table 1:** Seasonal adult mosquito abundance in Kilifi, Kisumu, and Nairobi between October 2014 and June 2016 using CO_2_-baited BG-Sentinel traps

	Kilifi			Kisumu			Nairobi		
Mosquito species	Long-rains	Short-rains	Dry season	Long-rains	Short-rains	Dry season	Long-rains	Short-rains	Dry season
*Aedes aegypti* ^a^	2235	581	113	2577	414	522	1071	180	76
*Aedes bromeliae* ^b^	6	5	0	0	5	0	13	0	0
*Aedes metallicus* ^b^	1	3	0	10	0	0	0	0	0
*Aedes tarsalis* ^b^	3	0	0	0	0	0	0	0	0
*Aedes dentatus*	0	1	0	0	0	0	0	0	0
*Aedes mcintoshi*	2	0	0	50	1	16	101	1	1
*Aedes tricholabis*	57	19	0	1	3	0	2295	18	6
*Aedes hirsutus*	23	3	0	0	1	0	0	0	0
*Aedes longipalpis*	33	0	0	0	0	0	0	0	0
*Aedes* spp.	109	1	0	19	0	0	0	39	6
*Eretmapodites chrysogaster* ^b^	5	6	0	0	0	0	2	0	0
*Mansonia africana*	2	0	0	789	39	185	0	0	0
*Mansonia uniformis*	0	0	0	224	7	220	0	0	0
*Mansonia* spp.	0	0	0	37	0	0	0	0	0
*Culex pipiens*	55	4	2	126	1	4	44	5	48
*Culex annuloris*	0	2	0	3	0	0	33	2	14
*Culex univittatus*	2	1	0	140	4	0	12	0	3
*Cx vansomereni*	3	0	0	0	0	0	21	0	0
*Culex rubinotus*	0	1	7	0	0	0	0	0	0
*Culex zombaensis*	0	0	0	0	0	0	54	0	10
*Culex tigripes*	0	0	0	1	0	2	3	0	0
*Culex poicilipes*	0	0	0	10	0	0	0	0	0
*Culex ethiopicus*	0	0	0	7	1	0	0	0	3
*Culex bitaeniorhynchus*	0	0	0	2	0	0	0	3	0
*Culex* spp.	7	2	0	23	0	0	0	2	2
*Toxorhynchites brevipalpis*	3	0	0	0	1	0	0	0	0
*Anopheles gambiae* (*s.l.*)	2	0	0	1	0	0	2	0	0
*Anopheles coustani*	0	0	0	1	0	0	0	0	0
Total	2548	629	122	4021	477	949	3651	250	169

^a^Major vector of DENV and urban YFV

^b^Potential YFV vectors

Total mosquito abundance was significantly higher in Kisumu than Kilifi (Estimate = 0.593 ± 0.29, *t* = 2.08, *P* = 0.043). However, total mosquito abundance did not differ between Kisumu and Nairobi (Estimate = 0.30 ± 0.27, *t* = 1.12, *P* = 0.27) or Kilifi and Nairobi (Estimate = 0.293 ± 0.30, *t* = 0.97, *P* = 0.34) (Table 2). Overall abundance during the long-rains was significantly higher than during the short-rains (Estimate = 2.316 ± 0.38, *t* = 5.459, *P* < 0.0001) and dry season (Estimate = 2.119 ± 0.39, *t* = 5.46, *P* < 0.0001), but collections between the short-rains and dry season did not differ significantly (Estimate = -0.198 ± 0.51, *t* = 0.39, *P* = 0.7) (Table 2).

**Table 2:** Total mosquito abundance and diversity in the long-rains, short-rains and dry season in Kilifi, Kisumu and Nairobi. Analyses are quasipoisson generalized linear model (Abundance *df *= 2, 47), normal linear models (Shannon diversity *df* = 2, 47). Kilifi was considered as the reference city and the dry season as the reference season in the analyses

	Total abundance			*Aedes agypti* abundance			*Aedes bromeliae* abundance			Shannon diversity index		
City	Estimate ± SE	*t*-value	*P-*value	Estimate ± SE	*t*-value	*P*-value	Estimate ± SE	*t*-value	*P*-value	Estimate ± SE	*t*-value	*P*-value
Kisumu	0.593 ± 0.285	2.083	0.043*	0.321 ± 0.241	1.33	0.19	-0.469 ± 0.803	-0.584	0.562	0.063 ± 0.035	1.821	0.075
Nairobi	0.293 ± 0.303	0.969	0.337	-0.653 ± 0.317	-2.058	0.045*	0.487 ± 0.615	0.792	0.432	0.186 ± 0.035	5.36	< 0.0001**
Long-rains	2.119 ± 0.388	5.459	< 0.0001**	2.109 ± 0.378	5.585	< 0.0001**	18,497 ± 2128	0.009	0.993	0.086 ± 0.037	2.303	0.026*
Short-rains	-0.198 ± 0.509	0.388	0.700	0.104 ± 0.455	0.229	0.82	17,497 ± 2128	0.008	0.993	-0.009 ± 0.035	-0.262	0.794

*P < 0.05, **P < 0.0001

*Aedes aegypti* accounted for 60% (*n* = 7772) of the total host-seeking mosquitoes collected, with Kilifi yielding 37.7% (*n* = 2932), Kisumu 45.2% (*n* = 3513), and Nairobi 17.1% (*n* = 1327) (Table 1). While *Ae. aegypti* abundance in Kilifi and Kisumu were comparable (Estimate = 0.321 ± 0.241, *t* = 1.33, *P* = 0.19), when each was compared to Nairobi, a two-fold and three-fold increase in *Ae. aegypti* abundance was observed in Kilifi (Estimate = -0.653 ± 0.32, *t* = -2.06, *P* = 0.045), and Kisumu (Estimate = 0.97 ± 0.31, *t* = 3.17, *P* = 0.027), respectively (Table 2). While *Ae. aegypti* abundance varied significantly between the long- and short-rains (Estimate = 2.004 ± 0.31, *t* = 6.50, *P* < 0.0001), and the long-rains and dry season (Estimate = 2.109 ± 0.378, *t* = 5.59, *P* < 0.0001), the numbers recorded in the short-rains and dry season were not significantly different (Estimate = 0.104 ± 0.46, *t* = 0.23, *P* = 0.82) (Table 2).

*Aedes bromeliae* was the second most dominant *Stegomyia* species recorded in all three cities, comprising 0.23% (*n* = 29) of the total mosquitoes collected, of which 37.9% (*n* = 11) occurred in Kilifi, 17.2% (*n* = 5) in Kisumu, and 44.8% (*n* = 13) in Nairobi*. Aedes bromeliae* abundance, however, did not vary by city or season (Table 2). Other potential vectors of YFV recorded include *Aedes metallicus*, *Aedes tarsalis* and *Erytmapodites chrysogaster*, although in very low numbers, each representing less than 0.1% of the total mosquitoes collected (Table 1). *Aedes metallicus*, *Ae. tarsalis* and *Er. chrysogaster* were all recorded in Kilifi, with no record of *Er. chrysogaster* and *Ae. metallicus* in Kisumu and Nairobi, respectively. The mosquito species composition encountered throughout the sampling periods in the different cities is shown in Table 1.

### Mosquito species richness/diversity

**Fig. 2:**
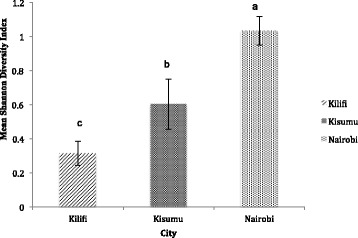
Mean Shannon diversity index for mosquitoes collected using BG-Sentinel traps in Kilifi, Kisumu and Nairobi in Kenya. Means followed by different letters are significantly different at α = 0.05

Of the total 25 species observed in all three areas, 10 belonged to *Culex* and nine to *Aedes* (Table 1). Mosquito species richness varied by city and season, being comparable in Kisumu and Nairobi (Estimate = 0.133 ± 1.03, *t* = 0.13, *P* = 0.90), but significantly higher when each was compared to Kilifi (Nairobi-Kilifi Estimate = 2.168 ± 0.96, *t* = 2.26, *P* = 0.03, and Kisumu-Kilifi Estimate = 2.301 ± 0.96, *t* = 2.40, *P* = 0.02). Mosquito species richness was significantly higher in the long-rains compared to the short-rains (Estimate = 5.77 ± 0.96, *t* = 6.02, *P* < 0.0001) and dry season (Estimate = 6.87 ± 1.03, *t* = 6.68, *P* < 0.0001), but not between the short-rains and dry season (Estimate = 1.098 ± 0.96, *t* = 1.15, *P* = 0.26). Species richness varied from 6 to 20 in Kilifi, 6 to 22 in Kisumu, and 10 to 18 species in Nairobi from the dry to rainy seasons. Also, the overall mosquito diversity varied by city and season. Mean mosquito diversity was two-fold higher in Nairobi (*H* = 1.03) compared to Kisumu (*H* = 0.60, Estimate = −0.123 ± 0.037, *t* = -3.30, *P* = 0.002), and three-fold higher in Nairobi compared to Kilifi (*H* = 0.31, Estimate = 0.186 ± 0.035, *t* = 5.36, *P* < 0.0001) (Table 2, Fig. 2). Mosquito diversity was, however, not significantly different between Kilifi and Kisumu (Estimate = 0.063 ± 0.035, *t* = 1.821, *P* = 0.075) (Table 2). We found significantly higher mosquito diversity in the long- versus short-rains (Estimate = 0.095 ± 0.035, *t* = 2.73, *P* = 0.009), than between the long-rains and dry season (Estimate = 0.086 ± 0.037, *t* = 2.303, *P* = 0.026), but no difference between the short-rains and dry season (Estimate = 0.009 ± 0.035, *t* = 0.26, *P* = 0.79) (Table 2). Mosquito diversity ranged from 0.04 to 1.9, with the lowest value recorded in Kisumu during the dry season and the highest in Nairobi in the dry season.

### Resting preferences

Of the 450 houses (150 per city, 50 per season) inspected from all three cities, 10% (*n* = 44) were positive for either male or female *Ae. aegypti*. Of these positive houses, 27.3% (*n* = 12) were from Kilifi, 52.3% (*n* = 23) from Kisumu and 20.4% (*n* = 9) from Nairobi. A total of 73 *Ae. aegypti* only were aspirated from all three cities, 44% (*n* = 32) females and 56% (*n* = 41) males both indoors and outdoors. This translated to a resting density of 0–5 mosquitoes indoors and 6–21 mosquitoes outdoors for the different cities (Table 3). Apart from one *Ae. bromeliae*, which was collected outdoors in Kilifi, *Ae. aegypti* was the only other *Stegomyia* species sampled in the resting collection. While there was no significant difference in the number of *Ae. aegypti* found resting indoors and outdoors in Kilifi (*χ* ^2^ < 0.0001, *df* = 1, *P* = 1.0), significantly higher numbers were found resting outdoors compared to indoors in Kisumu (*χ* ^2^ = 28.17, *df* = 1, *P* < 0.0001). In Nairobi, resting was exclusively outdoors. Also, in Kilifi, the catches of females (*χ* ^2^ = 0, *df* = 1, *P* = 1.0) either indoors or outdoors, like that of males (*χ* ^2^ = 1, *df* = 1, *P* = 0.32), did not differ significantly. However, in Kisumu significantly more females (*χ* ^2^ = 6.25, *df* = 1, *P* = 0.012) and males (*χ* ^2^ = 19.2, *df* = 1, *P* < 0.0001) were captured outdoors than indoors. Overall, in Kilifi the total number of females aspirated was significantly higher than the number of males (*χ* ^2^ = 6.55, *df* = 1, *P* = 0.011), while in Kisumu (*χ* ^2^ = 3.13, *df* = 1, *P* = 0.077) and Nairobi (*χ* ^2^ = 0, *df* = 1, *P* = 1), there was no significant difference.

**Table 3:** Indoor and outdoor resting densities of *Aedes aegypti* mosquitoes collected in Kilifi, Kisumu and Nairobi using Prokopack aspirators from October 2014-June 2016

			Female		Male		Total^a^	
Area	Location	Positive houses^b^	No. collected	Resting density^c^	No. collected	Resting density^c^	No. collected	Resting density^c^
Kilifi	Indoor^d^	3.3 (0.01–0.08*) (*n* = 5)	5	5	0	0	5	5
	Outdoor^e^	5.3 (0.03–0.11*) (*n* = 8)	7	4	4	2	11	6
Kisumu	Indoor	1.3 (0.002–0.05*) (*n* = 2)	1	1	1	1	2	2
	Outdoor	14.0 (0.09–0.21*) (*n* = 21)	14	7	28	14	42	21
Nairobi	Indoor	0 (0.0–0.03*) (*n* = 0)	0	0	0	0	0	0
	Outdoor	6.0 (0.03–0.11*) (*n* = 9)	5	3	8	4	13	7

^a^Total males and females *Ae. aegypti* collected

^b^% Positive houses (95% confidence interval) (*n*, number of positive houses)

^c^Resting density = No. collected/No. of collectors

^d^Indoors: sitting room, bedroom and kitchen

^e^Outdoor: nearby vegetation and outside walls

*P < 0.0001

## Discussion

*Aedes aegypti*, the known DENV vector in Kenya [9], was generally found to be the most abundant mosquito species. Abundance was highest during the long-rains, relative to the short-rains and dry season, and comparably higher in Kilifi and Kisumu *vs* Nairobi. We found very low occurrence of *Ae. bromeliae*, a species which did not vary by city or season. Furthermore, variation in mosquito diversity was evident, being highest in Nairobi and during the long-rains (Table 2). Variation in abundance and diversity, both important epidemiological risk parameters, may impact differentially on transmission risk of DENV/YFV across seasons and cities. More outdoor resting was observed for *Ae. aegypti*, suggesting the existence of an exophilic population especially in Kisumu and Nairobi. In Kilifi, resting data are suggestive of a more endophilic population of *Ae. aegypti*.

The *Ae. aegypti* abundance pattern was strongly correlated with seasonal rainfall, with higher abundance during the long-rains compared to the other periods, in all three sampling cities (Table 1). In fact, 5 and 8 times more *Ae. aegypti* were captured during the long-rains compared to short-rains and dry season, respectively (Table 1). This is expected, as the abundance of mosquitoes, including *Ae. aegypti*, is generally associated with rainfall [25]. Previous findings have found rainfall to be an important driver of *Ae. aegypti* populations and dengue incidence [25], corroborating the occurrence of dengue outbreaks in Kenya and East Africa during periods of heavy rainfall [9, 26]. Taken together, our findings suggest higher risk of DENV transmission in Kilifi/Kisumu than Nairobi, particularly during the long-rains. Nonetheless, the persistence of *Ae. aegypti* throughout the short-rains and dry season suggests that disease transmission could potentially persist throughout the year due to continued presence of the vector, albeit at lower levels.

Factors relating to availability of breeding sites, temperature or altitudinal differences may have influenced the abundance patterns of *Ae. aegypti* across the cities [25, 27]. Being a typical container breeder, we previously found an increase in the number of breeding sites in Kilifi and Kisumu in the long-rains, compared to Nairobi [17], which is located at a higher altitude (1661 masl), and has lower average monthly temperatures (22–28 °C), compared to Kilifi (50 masl, 27–31 °C) and Kisumu (1131 masl, 28–30 °C). This may partly explain the low *Ae. aegypti* abundance found in Nairobi, as significant reductions in *Ae. aegypti* abundance with an increase in altitude have previously been reported [27]. The same study identified temperature to be a positive risk factor for *Ae. aegypti* abundance, with vector abundance increasing with an increase in temperature [27]. Autochthonous cases of dengue can be facilitated by local *Aedes* vectors. However, despite Nairobi being a major hub in East Africa, there no outbreak of dengue has been reported. A possible contributing factor to this pattern could be low *Aedes* abundance, as was observed in our study, among other factors. The high vector abundance in Kilifi and Kisumu corroborates with their increased breeding sites, especially during the long-rains. This high *Ae. aegypti* abundance in Kilifi may explain the dengue epidemics reported in this region [7–10]. Also, the high abundance in Kisumu may explain the recent reports of sporadic cases of dengue (R. Sang, unpublished data), although outbreaks have not been reported. The high abundance of *Ae. aegypti* in Kisumu, and its potential role in dengue epidemics, is therefore deserving of further consideration.

*Aedes bromeliae*, *Ae. metallicus*, *Ae. tarsalis* and *Er. chrysogaster* are sylvatic vectors mostly found inhabiting discarded plastic containers as well as natural containers (tree holes, rock pools and discarded coconut shells) [17]. These YFV vectors have been implicated in yellow fever outbreaks in East and Central Africa [28–31]. Trap captures for these species were generally low and it is not certain if this could be related to potential sampling bias of the BG-Sentinel trap employed for sampling. This trap, whilst designed to target *Ae. aegypti* [32, 33], has been shown to effectively collect other disease vectors including *Anopheles* species and even sandflies [34, 35]. As such, it appears unlikely that this trapping tool may have affected the overall mosquito diversity and abundance, particularly as we also baited the traps with CO_2,_ which is thus far known to be the most potent attractant to mosquito species [36, 37]. However, given that in our previous study [17] we encountered high numbers of *Ae. bromeliae* and *Er. chrysogaster* immatures, the low numbers of adults recorded in this study suggest that adults may be poorly attracted to the BG-Sentinel trap. This indicates that developing better sampling tools for targeting adults of these species is an important consideration. In addition, the introduction of sylvatic YFV vectors into urban areas, as was observed in this study, is of public health concern. The YFV could adapt to these vectors given their ability to act as potential enzootic vectors. Their ability to transmit the YFV therefore warrants further assessment.

Although species richness was comparable in Nairobi and Kisumu, Nairobi had the highest overall diversity of mosquitoes (Fig. 2). The species observed in Kilifi, especially *Ae. bromeliae*, *Ae. metallicus*, *Ae. tarsalis* and *Er. chrysogaster*, which in addition to *Ae. aegypti*, are known vectors for YFV, should not be ignored. These species may be of epidemiological value, in the light of pathogen adaptation to multiple vectors as observed for chikungunya virus in Senegal [38, 39]. Their role in sustaining an outbreak of dengue and chikungunya therefore needs to be assessed, as these vectors, especially *Ae. bromeliae*, could serve as bridge vectors [40], moving the virus from the sylvatic to the urban transmission cycle. While mosquito diversity was higher during the rainy seasons compared to the dry season in Kilifi and Kisumu, the observed pattern was different in Nairobi, where higher mosquito diversity was recorded in the dry season. The diversity pattern in Nairobi may have been influenced by the *Culex* collections, which were fairly represented in Nairobi with only sparse occurrence in Kilifi/Kisumu, especially during the dry season. Although *Culex* species are not important vectors in the epidemiology of DENV/YFV, they play an important role in the transmission of other arboviruses, such as West Nile virus [41].

Despite extensive sampling effort, we found very low numbers of resting *Ae. aegypti* mosquitoes, both indoors and outdoors. The sampling regime and effort is in line with techniques employed elsewhere [42, 43]. The low numbers of *Ae. aegypti* resting indoors is in stark contrast to the largely indoor habit known for this species in Asia and South America [42, 44, 45]. This suggests a difference in the resting patterns of *Ae. aegypti* found in Kenya and corroborates findings from previous studies reporting generally low numbers of resting *Ae. aegypti* in this region [9, 46]. The largely outdoor resting habit of this species concurs with its breeding pattern being mainly outdoors [9, 17, 46]. However, the low outdoor numbers recorded here suggest other resting sites, apart from vegetation, that require further elucidation. Although most of the outdoor resting was observed on vegetation, this study did not investigate in detail the preferred plant types, as this was not within the scope of the study. Overall, the knowledge of these resting patterns can be exploited in emergency operations targeting adults to break transmission in an outbreak situation. The proportion of adults found resting indoor/outdoor varied between Kilifi and the other cities; for Nairobi and Kisumu it was largely outdoors. This finding indicates possible population differences in resting habits among these cities, which is worth exploring.

## Conclusions

*Aedes aegypti* was the most dominant mosquito species recorded and its occurrence varied by city and season. The abundance pattern suggests that the risk of DENV transmission is elevated during the long-rains and in Kilifi/Kisumu compared to Nairobi, assuming that the vector populations are similarly anthropophagic and efficient in transmitting the virus, but this is still under investigation. The low abundance of *Ae. bromeliae* recorded is suggestive of a low risk of YFV transmission in all three cities. The overall mosquito abundance pattern neither correlated with species richness nor diversity. In addition to vector abundance, feeding habits and vector competence are factors that can differentially drive the emergence of dengue and yellow fever in an area. Therefore, to fully understand the risk associated with the transmission of DENV/YFV in these cities of Kenya, these factors need to be assessed. The resting pattern for *Ae. aegypti* is suggestive of a more endophilic population in Kilifi, and an exophilic population in Kisumu and Nairobi. This knowledge on the resting behavior can be exploited in adult mosquito control to break transmission during emergency outbreak situations. Continuous vector surveillance should, however, be routinely performed for early detection of changing vector dynamics that may precipitate an outbreak of dengue/yellow fever.

## Abbreviations

DENV: dengue virus
GLM: general linear models
TEA: triethylamine
YFV: yellow fever virus
